# Expanding the horizon: Discovery of two new spider species of the *Pholcus phungiformes* species group (Araneae, Pholcidae) from Inner Mongolia, China, with a new western record for the group

**DOI:** 10.3897/zookeys.1275.186998

**Published:** 2026-04-02

**Authors:** Meichen Yan, Yating Liu, Luyu Wang, Zhiyuan Yao

**Affiliations:** 1 College of Life Science, Shenyang Normal University, Shenyang 110034, Liaoning, China Southwest University Chongqing China; 2 Key Laboratory of Eco-environments in Three Gorges Reservoir Region (Ministry of Education), School of Life Sciences, Southwest University, Chongqing 400715, China Shenyang Normal University Shenyang China

**Keywords:** Biodiversity, cellar spider, daddy-long-legs spider, East Asia, invertebrate, new record, new species, taxonomy, zoogeographic range

## Abstract

The spiders of the *Pholcus phungiformes* species group are primarily distributed across five mountain ranges in East Asia: the Lüliang Mountains and the Yanshan-Taihang Mountains in North China, the transitional uplands between the Yanshan-Taihang and Changbai Mountains, the Changbai Mountains straddling the China-North Korea border, and the Taebaek-Sobaek Mountains on the Korean Peninsula. In this study, we report the first records of this group from Inner Mongolia, northern China, describing two new species: *Pholcus alashan* Wang & Yao, **sp. nov**. (♂♀, from Alashan Zuoqi) and *P. chifeng* Yan & Yao, **sp. nov**. (♂♀, from Chifeng). The discovery of *P. alashan***sp. nov**. extends the group’s western distribution limit by 5.2° longitude (from c. 111.0°E to 105.8°E), representing a significant zoogeographic range expansion.

## Introduction

*Pholcus* Walckenaer, 1805 is the most diverse genus within the family Pholcidae C.L. Koch, 1850. It is predominantly distributed across the Afrotropical, Palaearctic, Indo-Malayan, and Australasian regions (Huber 2011; Yao and Li 2012, 2025; WSC 2026). This genus encompasses 21 species groups and 434 valid species (Huber 2011; Huber et al. 2018; WSC 2026). The *Pholcus phungiformes* species group is the most species-rich, with 140 species (Huber 2011; Wang et al. 2020; Yao et al. 2021; Lu et al. 2022; Zhao et al. 2023a, 2023b; Lee et al. 2024; Li et al. 2025). Almost all species within this group have been recorded from five mountain ranges: the Lüliang Mountains (9 spp.) and the Yanshan-Taihang Mountains (35 spp.) in North China, the mountainous regions (6 spp.) located between the Yanshan-Taihang and Changbai Mountains, the Changbai Mountains (28 spp.) at the border between northeastern China and North Korea, as well as the Taebaek-Sobaek Mountains (62 spp.) on the Korean Peninsula. The only exception is *P. phungiformes* Oliger, 1983, which has been reported from the Maritime Territory, Sakhalin Island, and the Kurile Islands in Russia (Huber 2011). The records from the Lüliang Mountains represent the westernmost distribution limit of the *P. phungiformes* species group.

**Figure 1.**
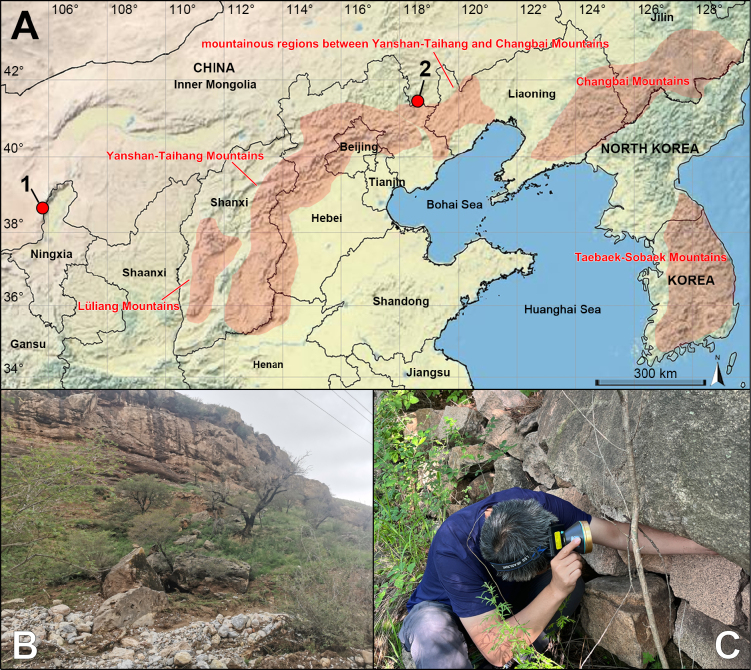
Distribution records and habitats of new species within the *Pholcus phungiformes* species group. **A**. Distribution records (**1**. *P. alashan* sp. nov.; **2**. *P. chifeng* sp. nov.); **B**. Habitat of *P. alashan* sp. nov.; **C**. Habitat of *P. chifeng* sp. nov.

In China, a total of 77 species within the *P. phungiformes* species group have been documented, representing 55% of the global total for this group (Zhang et al. 2025; WSC 2026). During our examination of specimens collected from Inner Mongolia, northern China, in 2025, we identified two previously undescribed species belonging to this group. One was discovered in Chifeng, while the other was found in Alashan Zuoqi, marking the westernmost extent of this group’s distribution (Fig. 1).

## Materials and methods

Specimens were examined and measured with a Leica M205 C stereomicroscope in the laboratory of the Shenyang Normal University (**SYNU**) in Liaoning, China. Left male palps were photographed. Epigynes were photographed before dissection. Vulvae were photographed after treatment in a warm 10% solution of potassium hydroxide (KOH) to dissolve soft tissues. Images were captured with a Canon EOS 750D wide zoom digital camera (24.2 megapixels) mounted on the stereomicroscope mentioned above and assembled using Helicon Focus v. 3.10.3 image stacking software (Khmelik et al. 2005). All measurements are given in millimeters (mm). Leg measurements are shown as: total length (femur, patella, tibia, metatarsus, tarsus). Leg segments were measured on their dorsal side. The distribution map was generated with ArcGIS v. 10.2 (ESRI Inc.). The specimens studied are preserved in 75% ethanol and are deposited in the College of Life Science, Shenyang Normal University.

Terminology and taxonomic descriptions follow Huber (2011) and Yao et al. (2015, 2021). The following abbreviations are used: **aa** = anterior arch, **ALE** = anterior lateral eye, **AME** = anterior median eye, **b** = bulb, **da** = distal apophysis, **dp** = distal process, **ds** = dorsal spine, **dsa** = dorso-subdistal apophysis, **e** = embolus, **fa** = frontal apophysis, **kn** = knob, **L/d** = length/diameter ratio, **ml** = membranous lamella, **pa** = proximo-lateral apophysis, **PME** = posterior median eye, **pp** = pore plate, **pr** = procursus, **pra** = proximal apophysis, **pvp** = prolatero-ventral protrusion, **rpa** = retrolatero-proximal apophysis, **sa** = subdistal apophysis, **u** = uncus, **va** = ventral apophysis, **vda** = ventro-distal apophysis, **vp** = ventral protrusion.

## Taxonomic accounts

### Family Pholcidae C.L. Koch, 1850

**Subfamily Pholcinae C.L. Koch, 1850**

#### Genus Pholcus

Taxon classificationAnimaliaAraneaePholcidae

Walckenaer, 1805

83ECF929-0AE6-5E4B-A44C-A964B4CA4F34

##### Type species.

*Aranea phalangioides* Fuesslin, 1775.

### *Pholcus phungiformes* species group

**Remarks**. This species group was recognized by Huber (2011). The two new species described below are assigned to this group by the following combination of characters: male chelicerae with frontal apophyses (fa), male palpal tibia with a prolatero-ventral protrusion (pvp), uncus with a “pseudo-appendix”, and epigyne with a knob (kn) (Li et al. 2025).

#### Pholcus alashan

Taxon classificationAnimaliaAraneaePholcidae

Wang & Yao sp. nov.

CF80FCBB-76C1-557E-87B7-D84A3C8939F2

https://zoobank.org/62C4B065-012E-4123-A57F-78EC55990207

Figs 2, 3

##### Type material.

***Holotype***: China • ♂; Inner Mongolia, Alashan Zuoqi, Helanshan Mountain, Guangzong Temple; 38.665300°N, 105.807267°E; alt. 1976 m; 9 June 2015; T. Lu, G. Huang, L. Wang et al. leg.; SYNU-Ar00534. ***Paratypes***: China • 3♀; same locality and collectors as for the holotype; 8 June 2015; SYNU-Ar00535–537 • 3♂; same locality as for the holotype; 8 August 2025; Z. Yao, B. Wang & X. Li leg.; SYNU-Ar00538–540 • 3♀; same data as for preceding; SYNU-Ar00541–543.

##### Etymology.

The specific name refers to the type locality; noun in apposition.

##### Diagnosis.

**Figure 2.**
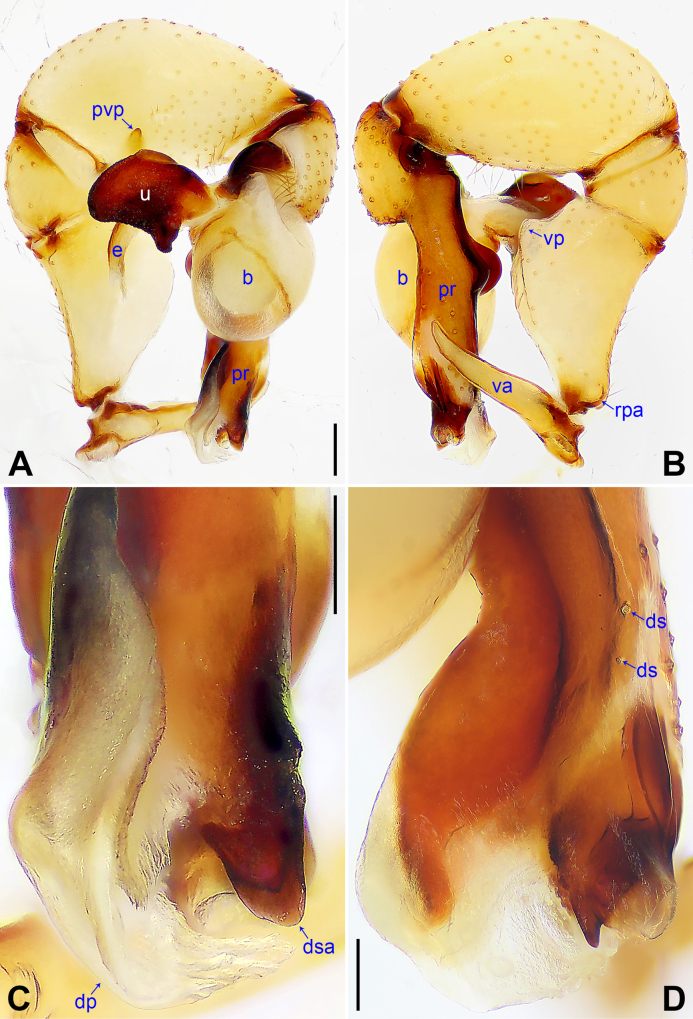
*Pholcus alashan* sp. nov., holotype male. **A**. Palp, prolateral view; **B**. Palp, retrolateral view; **C**. Distal part of procursus, prolateral view; **D**. Distal part of procursus, dorsal view. Abbreviations: b = bulb, dp = distal process, ds = dorsal spine, dsa = dorso-subdistal apophysis, e = embolus, pr = procursus, pvp = prolatero-ventral protrusion, rpa = retrolatero-proximal apophysis, u = uncus, va = ventral apophysis, vp = ventral protrusion. Scale bars: 0.20 (**A, B**); 0.10 (**C, D**).

**Figure 3.**
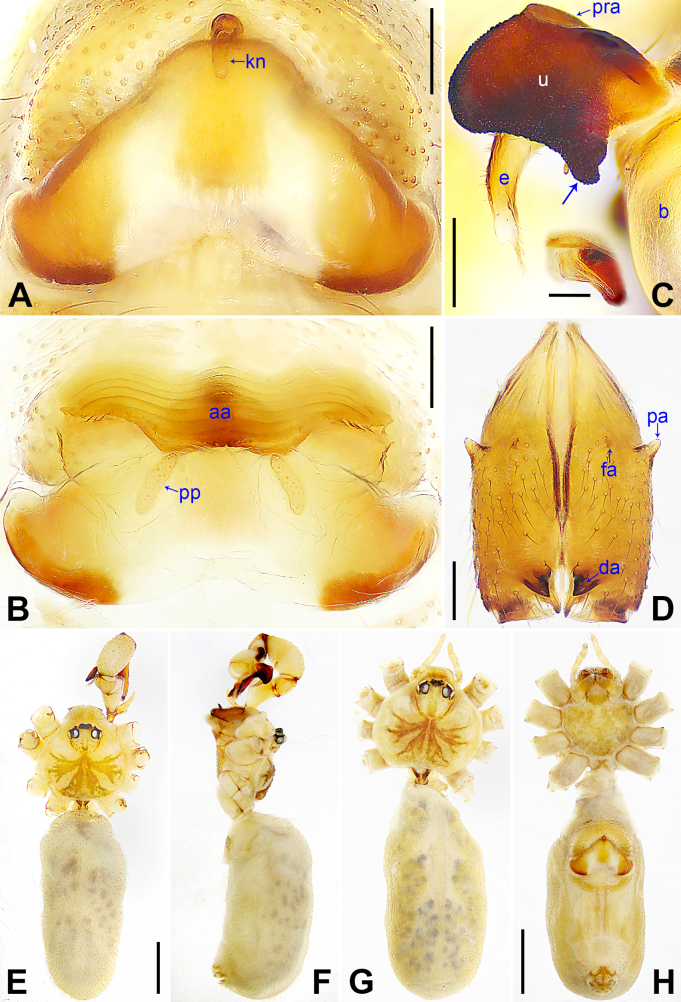
*Pholcus alashan* sp. nov., holotype male (**C–F**) and paratype female (**A, B, G, H**). **A**. Epigyne, ventral view; **B**. Vulva, dorsal view; **C**. Bulbal apophyses, prolateral view (arrow indicates strongly protruding part; the insert is retrolateral view of “pseudo-appendix”); **D**. Chelicerae, frontal view; **E, G**. Habitus, dorsal view; **F**. Habitus, lateral view; **H**. Habitus, ventral view. Abbreviations: aa = anterior arch, b = bulb, da = distal apophysis, e = embolus, fa = frontal apophysis, kn = knob, pa = proximo-lateral apophysis, pp = pore plate, pra = proximal apophysis, u = uncus. Scale bars: 0.20 (**A–D**); 0.10 (the insert in **C**); 1.00 (**E–H**).

The new species can be easily distinguished from all known congeners in the *P. phungiformes* species group by the following combination of characters: procursus with curved distal membranous process (dp in Fig. 2C) and sclerotized dorso-subdistal apophysis (dsa in Fig. 2C), uncus proximo-laterally strongly protruding (arrow in Fig. 3C), epigyne nearly triangular (Fig. 3A), and vulval pore plates as long as epigynal knob (pp in Fig. 3B, kn in Fig. 3A).

##### Description.

**Male (*holotype*): *Measurements***: Total length 5.31 (5.51 with clypeus), carapace 1.55 long, 1.88 wide, opisthosoma 3.76 long, 1.68 wide. ***Leg I***: 39.48 (10.06, 0.74, 10.13, 16.15, 2.40), ***leg II***: 27.36 (7.56, 0.72, 6.79, 10.77, 1.52), ***leg III***: 19.51 (5.71, 0.64, 4.68, 7.44, 1.04), ***leg IV***: 26.14 (7.76, 0.66, 6.44, 9.94, 1.34); ***tibia I L/d***: 56. ***Eye interdistances and diameters***: PME–PME 0.24, PME 0.15, PME–ALE 0.04, AME–AME 0.05, AME 0.12. ***Sternum width/length***: 1.29/1.05.

***Color***: Carapace yellowish, with radiating marks; ocular area yellowish, with median and lateral brown bands; clypeus yellowish; sternum yellowish, with brown marks. Legs yellowish, but whitish on distal parts of femora and tibiae, without darker rings on femora and tibiae. Opisthosoma yellowish, with dorsal and lateral spots.

***Body***: As in Fig. 3E, F; ocular area elevated, without eye-stalks.

***Chelicerae***: As in Fig. 3D, with pair of proximo-lateral apophyses (pa), pair of distal apophyses (da) with two teeth each, and pair of frontal apophyses (fa).

***Legs***: Retrolateral trichobothrium on tibia I situated at 3% proximally; legs with short vertical setae on tibiae, metatarsi, and tarsi; tarsus I with 38 distinct pseudosegments.

***Palp***: As in Fig. 2A, B; trochanter with long (5× longer than wide) ventral apophysis (va); femur with retrolatero-proximal apophysis (rpa) and distinct ventral protrusion (vp); tibia with prolatero-ventral protrusion (pvp); procursus (pr) simple proximally, but complex distally, with raised prolatero-subdistal edge bearing distal membranous process (dp), sclerotized dorso-subdistal apophysis (dsa), and two dorsal spines (ds); uncus (u) with proximal apophysis (pra) and scales, proximo-laterally strongly protruding (arrow in Fig. 3C); “pseudo-appendix” semi-transparent (Fig. 3C); embolus (e) weakly sclerotized, with transparent distal projections.

**Female** (***paratype***, SYNU-Ar00535): Similar to male, habitus as in Fig. 3G, H. Total length 4.95 (5.18 with clypeus), carapace 1.35 long, 1.70 wide, opisthosoma 3.60 long, 1.64 wide; ***tibia I***: 6.86; ***tibia I L/d***: 43. ***Eye interdistances and diameters***: PME–PME 0.21, PME 0.16, PME–ALE 0.04, AME–AME 0.04, AME 0.09. ***Sternum width/length***: 1.09/0.93. Clypeus with brown marks. Epigyne (Fig. 3A) nearly triangular, posteriorly strongly curved, with knob (kn). Vulva (Fig. 3B) with sclerotized wave-shaped anterior arch (aa) and pair of long, elliptical pore plates (pp).

##### Variation.

Tibia I in three male paratypes (SYNU-Ar00538–540): 9.94, 10.51, 11.03. Tibia I in the other three female paratypes (SYNU-Ar00541–543): 7.88, 8.08, 8.64 (Leg I missing in SYNU-Ar00536–537).

##### Habitat.

Underside of overhang on rocky cliffs in a mountainous area.

##### Distribution.

China (Inner Mongolia, known only from the type locality; Fig. 1).

#### Pholcus chifeng

Taxon classificationAnimaliaAraneaePholcidae

Yan & Yao sp. nov.

3F2F7280-2421-5A11-80B0-683C0AF0D4AF

https://zoobank.org/57F0770B-D269-42CE-9068-3BA6DCD8DC59

Figs 4, 5

##### Type material.

***Holotype***: China • ♂; Inner Mongolia, Chifeng, Ningcheng County, Heilihe Town, Canglonggu Scenic Spot; 41.367897°N, 118.647410°E; alt. 859 m; 10 August 2025; Z. Yao, B. Wang & X. Li leg.; SYNU-Ar00544. ***Paratypes***: China • 1♂; same data as for the holotype; SYNU-Ar00545 • 3♀; same data as for the holotype; SYNU-Ar00546–548.

##### Etymology.

The specific name refers to the type locality; noun in apposition.

##### Diagnosis.

**Figure 4.**
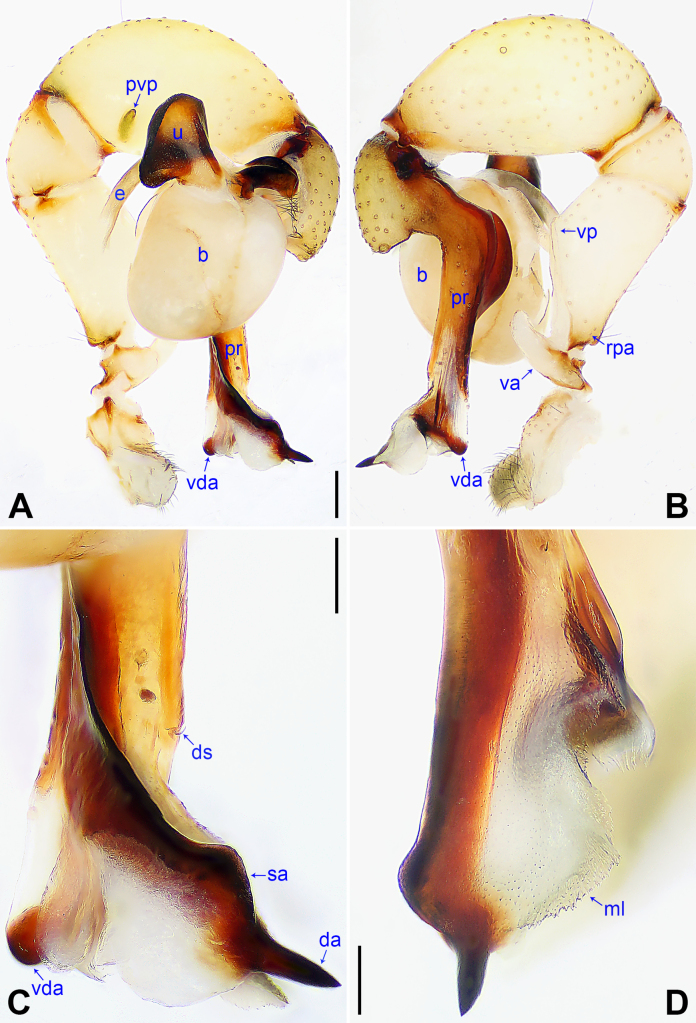
*Pholcus chifeng* sp. nov., holotype male. **A**. Palp, prolateral view; **B**. Palp, retrolateral view; **C**. Distal part of procursus, prolateral view; **D**. Distal part of procursus, dorsal view. Abbreviations: b = bulb, da = distal apophysis, ds = dorsal spine, e = embolus, ml = membranous lamella, pr = procursus, pvp = prolatero-ventral protrusion, rpa = retrolatero-proximal apophysis, sa = subdistal apophysis, u = uncus, va = ventral apophysis, vda = ventro-distal apophysis, vp = ventral protrusion. Scale bars: 0.20 (**A, B**); 0.10 (**C, D**).

**Figure 5.**
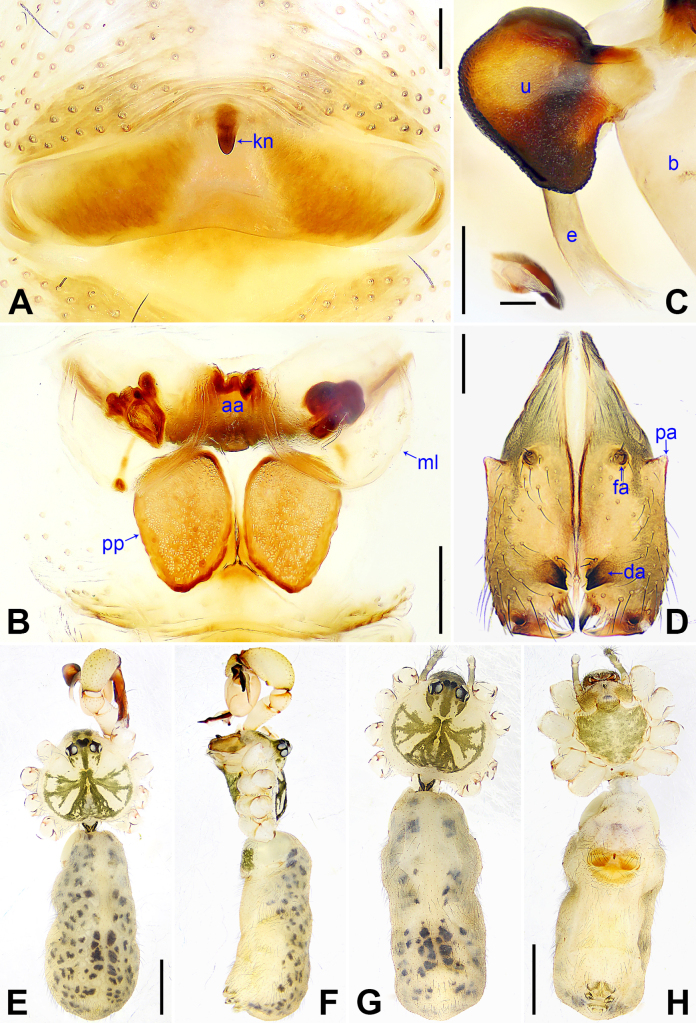
*Pholcus chifeng* sp. nov., holotype male (**C–F**) and paratype female (**A, B, G, H**). **A**. Epigyne, ventral view; **B**. Vulva, dorsal view; **C**. Bulbal apophyses, prolateral view (the insert is retrolateral view of “pseudo-appendix”); **D**. Chelicerae, frontal view; **E, G**. Habitus, dorsal view; **F**. Habitus, lateral view; **H**. Habitus, ventral view. Abbreviations: aa = anterior arch, b = bulb, da = distal apophysis, e = embolus, fa = frontal apophysis, kn = knob, ml = membranous lamella, pa = proximo-lateral apophysis, pp = pore plate, u = uncus. Scale bars: 0.10 (**A**); 0.20 (**B–D**); 0.10 (the insert in **C**); 1.00 (**E–H**).

The new species resembles *Pholcus liaoning* S. Li & Yao, 2025 (Li et al. 2025: 301, figs 11, 12) by having similar uncus, male chelicerae, and epigyne (Fig. 5A, C, D), but it can be distinguished by prolatero-subdistal edge of procursus with blunt subdistal apophysis (sa in Fig. 4C vs. absent), by procursus with blunt ventro-distal apophysis (vda in Fig. 4A–C vs. pointed), by vulval anterior arch bearing pair of membranous lamella with medially sclerotized part each (ml in Fig. 5B vs. absent), and by vulval pore plates anteriorly blunt (pp in Fig. 5B vs. pointed).

##### Description.

**Male (*holotype*): *Measurements***: Total length 5.18 (5.29 with clypeus), carapace 1.58 long, 1.86 wide, opisthosoma 3.60 long, 1.63 wide. ***Legs I and II*** missing, ***leg III***: 20.91 (6.06, 0.68, 5.06, 7.95, 1.16), ***leg IV***: 27.26 (7.82, 0.69, 6.79, 10.38, 1.58). ***Eye interdistances and diameters***: PME–PME 0.24, PME 0.15, PME–ALE 0.05, AME–AME 0.05, AME 0.09. ***Sternum width/length***: 1.23/1.05.

***Color***: Carapace yellowish, with brown radiating marks and marginal brown bands; ocular area yellowish, with median and lateral brown bands; clypeus and sternum yellowish, with brown marks. Legs yellowish, but dark brown on patellae and whitish on distal parts of femora and tibiae, with darker rings on subdistal parts of femora and proximal and subdistal parts of tibiae. Opisthosoma yellowish, with dorsal and lateral spots.

***Body***: As in Fig. 5E, F; ocular area elevated, without eye-stalks.

***Chelicerae***: As in Fig. 5D, with pair of proximo-lateral apophyses (pa), pair of distal apophyses (da) with two teeth each, and pair of frontal apophyses (fa).

***Legs***: Legs with short vertical setae on tibiae, metatarsi, and tarsi.

***Palp***: As in Fig. 4A, B; trochanter with short (2× longer than wide), retrolatero-proximally bulged ventral apophysis (va); femur with retrolatero-proximal apophysis (rpa) and indistinct ventral protrusion (vp); tibia with prolatero-ventral protrusion (pvp); procursus (pr) simple proximally, but complex distally, with raised prolatero-subdistal edge bearing pointed distal apophysis (da) and blunt subdistal apophysis (sa), ventro-distal apophysis (vda), dorso-distal membranous lamella (ml), and one slender dorsal spine (ds); uncus (u) nearly elliptical, with scales; “pseudo-appendix” semi-transparent (Fig. 5C); embolus (e) weakly sclerotized, with transparent distal projections.

**Female** (***paratype***, SYNU-Ar00546): Similar to male, habitus as in Fig. 5G, H. Total length 4.94 (5.05 with clypeus), carapace 1.46 long, 1.64 wide, opisthosoma 3.48 long, 1.61 wide; ***tibia I***: 7.44; ***tibia I L/d***: 47. ***Eye interdistances and diameters***: PME–PME 0.20, PME 0.15, PME–ALE 0.04, AME–AME 0.03, AME 0.08. ***Sternum width/length***: 1.02/0.90. Clypeus brown. Epigyne (Fig. 5A) posteriorly slightly curved, with knob (kn). Vulva (Fig. 5B) with ridge-shaped anterior arch (aa) bearing pair of membranous lamellae (ml) with medially sclerotized parts each, and pair of nearly quadrilateral pore plates (pp).

##### Variation.

Tibia I in male paratype (SYNU-Ar00545): 9.68; tibia I L/d: 69; retrolateral trichobothrium on tibia I situated at 3% proximally; tarsus I with 31 distinct pseudosegments. Tibia I in the other two female paratypes (SYNU-Ar00547–548): 7.83, 8.01.

##### Habitat.

Underside of overhang on rocky cliffs in a mountainous area.

##### Distribution.

China (Inner Mongolia, known only from the type locality; Fig. 1).

## Supplementary Material

XML Treatment for Pholcus

XML Treatment for Pholcus alashan

XML Treatment for Pholcus chifeng

## Additional information

### Use of AI

No use of AI was reported.

### Author ORCIDs

Meichen Yan https://orcid.org/0009-0001-7306-3323

Yating Liu https://orcid.org/0009-0009-5219-8745

Luyu Wang https://orcid.org/0000-0002-5250-3473

Zhiyuan Yao https://orcid.org/0000-0002-1631-0949

### Data availability

All of the data that support the findings of this study are available in the main text.
